# Renovascular Compression by the Diaphragmatic Crus: A Case Report

**DOI:** 10.7759/cureus.24004

**Published:** 2022-04-10

**Authors:** Ali Al-Smair, Osama Saadeh, Ahmad Saadeh, Ahmad Al-Ali

**Affiliations:** 1 Radiology, Medray International Radiology Center, Amman, JOR; 2 Public Health, Northeastern Illinois University, Chicago, USA; 3 Medicine, The University of Jordan, Amman, JOR; 4 Radiology, Jordan Ministry of Health, Amman, JOR

**Keywords:** case report, medical treatment, renal artery stenosis, renovascular hypertension, diaphragm crus

## Abstract

Renovascular hypertension (RVHT) is among the most prevalent causes of treatment-resistant hypertension. Mostly it is caused by renal artery stenosis (RAS). With atherosclerosis being the most common cause of RAS, RAS due to external compression by the diaphragmatic crus is rare. The treatment of rare causes requires individualization due to the differences between their etiologies. Herein, we present a case of an 18-year-old patient presenting with high blood pressure readings. On follow-up, he was diagnosed with hypertension. On further evaluation, the right diaphragmatic crus compressed the right renal artery.

This case emphasizes medical management in patients with hypertension secondary to diaphragmatic crus compression, and radiological findings in such cases.

## Introduction

When the topic of secondary hypertension is discussed, renovascular hypertension (RVHT) is always there, being one of the most common causes often leading to resistant hypertension. The cost of caring for uncontrolled hypertension is $48.6 billion each year [1], and 10% of them have secondary hypertension [2]. Furthermore, 75% of secondary hypertension cases have RVHT as the underlying etiology [3].

RVHT affects anyone at any given age and is commonly caused by fibromuscular dysplasia, extrinsic compression of a renal artery, renal artery dissection [3], arteritides, and renal artery stenosis (RAS) secondary to atherosclerosis [4]. RAS is the most common cause and can be seen in adults of age 65 and above. The prevalence of the RAS is higher in patients with atherosclerotic disease, while autopsy studies stated that “greater than 25% of all patients who die of cardiovascular disease have some degree of RAS” [4].

RAS resulting from external compression by the diaphragmatic crus is rare. As a result of that, when it comes to treating RAS cases that are caused by the external compression by the diaphragmatic crus, requires an individualized treatment plan. Meaning that each case presented should be studied individually and given the necessary treatment [5]. In this case, we will report a patient with high blood pressure that was caused by external compression by the diaphragmatic crus.

## Case presentation

**Figure 1:**
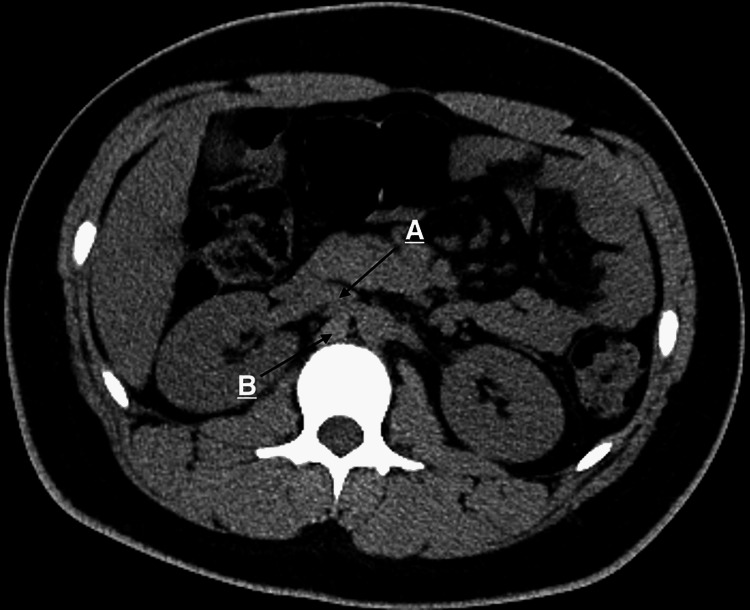
An axial CT scan. A - The right renal artery. B - A right hypertrophied diaphragmatic crus indenting the proximal right renal artery.

An 18-year-old patient presented to our clinic complaining of high blood pressure readings picked up at home. He has no previous medical history apart from smoking. Physical examination was normal, BMI of 26, ECG was normal but blood pressure was 145/95. After multiple readings in multiple settings within a week, readings were (140-148 over 92-97 mmHg) and he was diagnosed with hypertension. Labs were ordered to assess for secondary causes of hypertension, including hyperaldosteronism (renin-aldosterone ratio), pheochromocytoma (24-h urine metanephrine level, serum metanephrines), kidney function (creatinine), thyroid disease (thyroid function test), parathyroid hormone, and other routine labs including a lipid profile, glucose levels, electrolytes, and others which all were normal. He had no family history of secondary hypertension. He was started on Diltiazem and referred to a radiology clinic for an abdominal Doppler ultrasound and computed tomographic (CT) scan to rule out other causes. On further follow-ups, the blood pressure was not controlled on Diltiazem, and it was switched to angiotensin receptor blocker (ARB) Losartan. Abdominal Doppler ultrasound indicated stenosis in the right artery. The CT scan showed normal adrenal glands and an indentation of the diaphragmatic crus on the posterior aspect of the right renal artery. Other abdominal organs were completely normal (Figure 1).

**Figure 2:**
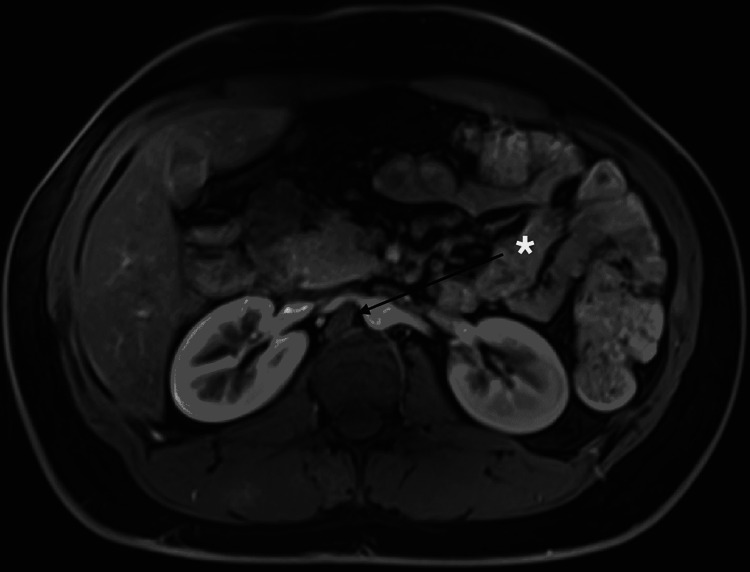
An axial postcontrast T1W fat sat showing a right hypertrophied diaphragmatic crus indenting the proximal right renal artery.

A magnetic resonance imaging (MRI) was ordered for better visualization of the diaphragm. It also showed normal abdominal organs, including the adrenals and the diaphragm abnormality (Figure 2).

The diagnosis of secondary hypertension due to compression of the right renal artery by the right diaphragmatic crus was confirmed. On regular follow-ups, blood pressure readings were normal or slightly elevated. The patient was informed about the treatment options regarding his case. The medical and surgical interventions were explained. However, he refused to have the surgery. Also, hydrochlorothiazide was added for better blood pressure control. On follow up, renal function test was initially slightly increased after starting the combination treatment, but it went back to normal at one-month follow-up. The patient was doing well on a one-year follow-ups, and blood pressure readings were within the normal range. 

## Discussion

RVHT is commonly caused by RAS. RAS is mostly caused by atherosclerosis followed by fibromuscular dysplasia to a lesser extent [5]. Its prevalence varies from 10 to 45% of refractory hypertension patients, and it is a correctable cause of RVHT [6]. Among various causes of RVHT, external compression of the renal artery by the diaphragmatic crus is a very rare entity [5,6]. Due to its rarity, literature describing hypertension secondary to diaphragmatic compression on the renal vessels is limited, with only 23 cases reported by a recent review. The review also showed that among cases reported, almost 48% (11) had left-sided compression, almost 17% (4) had right-sided compression, 13% (3) cases were bilateral, and the rest were unknown [7]. Our patient had a right-sided renal artery indentation by the diaphragmatic crus.

RVHT can vary in severity, it falls in a spectrum from mild symptoms to severe, to even vascular congestion and kidney failure. A severe decrease in renal perfusion induces a severe elevation in the blood pressure escalating end-organ damage [8]. RVHT secondary to an external compression is usually associated with abdominal aortic aneurysm, tumors, and others. Clinically features promoting RAS investigations are abdominal bruits, hypokalemia of unknown cause, retinopathy, and renal impairment. Kidney failure is one of the consequences of RAS. Hence, early detection and treatment are vital [9]. Our case presented with an incidental finding of increased blood pressure (145/95 mmHg). On one-week follow-up, home readings during that week averaged (140-148 over 92-97 mmHg). He was diagnosed with hypertension, and investigations were started to pinpoint the cause. Lab tests for hyperaldosteronism, pheochromocytoma, thyroid, parathyroid hormones, lipid profile, electrolytes, glucose, and others all were normal.

Radiological modalities have an important role in diagnosing anatomical causes of RAS. Doppler ultrasound is recommended to start with, it is a noninvasive and safe modality. However, it is an operator-dependent modality [10]. CT angiography and MR angiography are the preferred modalities for RAS, with 94% sensitivity and 99% specificity and 90% sensitivity and 86% specificity, respectively, in atherosclerosis and fibromuscular associated abnormalities [11]. CT scan and MRI also showed practicality in diagnosing and assessing relations between the diaphragm and renal artery [12]. Our patient had a Doppler ultrasound indicating stenosis greater than 60%. A CT scan and MRI showed normal abdominal organs, in addition to that, they showed the diaphragmatic crus compressing on the right renal artery, which in light of inconclusive investigations, confirms the diagnosis.

Treatment methods vary from medications to surgery, minimally invasive to open surgery. Medications used in RVHT angiotensin-converting enzyme inhibitor (ACEI)/ARB are the best options to start with. It showed an improvement in the survival rate [13]. Although ARB decreases the glomerular filtration rate (GFR) initially, it does not cause a long-term loss in renal function. In addition to that, ARB is a reno-protective drug [14,15]. A combination of ACE/ARB with calcium channel blockers and diuretics can attain the goal of blood pressure [15]. Renal revascularization endovascular or open are the surgical treatment method used in RVHT, with a preference for the endovascular method. Young aged patients with the non-atherosclerotic renovascular disease and others are among the factors favoring renal revascularization over medical treatment [13]. Despite the use of renal revascularization and surgery in the management of renal artery compression, they are associated with surgical complications and stent complications, and it has to be individualized. Muscular compression increases the risk for stent complications, and the movement of the diaphragm may displace it or bend it, leading to restenosis. Other modalities have been described, such as balloon angioplasty. It showed less effectiveness compared to stenting but with fewer complications [9]. An alternative new treatment with CT-guided botulinum toxin injection has been described for the compression by the diaphragmatic crus [16]. Our patient was first started on a calcium channel blocker (diltiazem), which had a minimum control on the blood pressure. Then it was switched to ARB (losartan), which did not achieve optimal control. Hydrochlorothiazide was added. Fortunately, the blood pressure readings were within the normal range. The patient was kept on the current regimen due to sufficient control of the blood pressure and adherence by the patient.

## Conclusions

RVHT is commonly caused by RAS. RAS is mainly caused by atherosclerosis and, to a lesser extent, fibromuscular dysplasia. There are many causes of secondary hypertension that should be ruled out. Lab tests and images are used to reach the diagnosis or to confirm it. Doppler ultrasound is a safe and non-invasive modality to assess for RAS. CT and MRI help in ruling out anatomical abnormalities, and intra-abdominal masses that may lead to hypertension. Treatment varies depending on the cause itself, in some cases, medical treatment could be sufficient to control blood pressure, whereas surgical treatment is the most effective and often curative, especially in anatomical abnormalities.
